# Homologous Repair‐Deficient Pancreatic Cancer: Refined Targeting of DNA Damage Response is an Effective Therapeutic Strategy

**DOI:** 10.1002/ueg2.12773

**Published:** 2025-08-18

**Authors:** Alica K. Beutel, Christopher J. Halbrook, Menar Ekizce, Jessica Lindenmayer, Elodie Roger, Sabrina E. Calderon, Thomas Seufferlein, Alexander Kleger, Johann Gout, Lukas Perkhofer

**Affiliations:** ^1^ Department of Internal Medicine I University Hospital Ulm Ulm Germany; ^2^ Department of Molecular Biology and Biochemistry University of California Irvine Irvine California USA; ^3^ Chao Family Comprehensive Cancer Center University of California Irvine Orange California USA; ^4^ Institute of Molecular Oncology and Stem Cell Biology Ulm University Hospital Ulm Germany; ^5^ Core Facility Organoids Ulm University Ulm Germany; ^6^ Division of Interdisciplinary Pancreatology Department of Internal Medicine I Ulm University Hospital Ulm Germany

**Keywords:** ATM serine/threonine kinase (ATM), breast cancer gene 1/2 (BRCA1/2), homologous recombination deficiency (HRD), pancreatic ductal adenocarcinoma (PDAC), PARP inhibitor (PARPi), partner and localizer of BRCA2 (PALB2), personalized medicine, talazoparib, targeted therapy

## Abstract

**Background:**

Pancreatic ductal adenocarcinoma (PDAC) is a devastating malignancy with a high mortality rate. While up to 20% of PDAC patients harbor mutations in genes involved in homologous recombination (HR) repair, only 5% of germline *BRCA1/2* mutation carriers have an approved treatment option with the PARP inhibitor (PARPi) olaparib. Characterizing HR‐deficient (HRD) genotypes beyond g*BRCA1/2* that are susceptible to PARPi has potential to substantially broaden the eligible patient population, and defining the optimal inhibitor may further optimize treatment strategies to advance personalized medicine in PDAC.

**Objective:**

Our previous preclinical work showed synthetic lethality of a multi‐pronged DNA damage repair interference strategy using the PARPi olaparib, ATR inhibitor VE‐822, and DNA‐PK inhibitor CC‐115 (termed PAD) in ATM deficiency. In the present study, we challenged the role of olaparib in our PAD combination and assessed the regimen's efficacy across various HRD genotypes.

**Methods:**

We assessed a spectrum of DNA damage repair‐interfering drugs to identify the most potent inhibitor in HRD. Using *ATM*, *BRCA1*, *BRCA2* and *PALB2*‐defective versus HR‐proficient murine PDAC cells, we systematically investigated the feasibility of expanding an optimized PAD regimen within defined HRD genotypes in vitro and in vivo. The regimen's efficacy was validated in PDAC patient‐derived organoids with and without deleterious class IV/V alterations in HRD genes.

**Results and Conclusion:**

Here, we demonstrate a remarkable potency of the PARPi talazoparib in HRD PDAC. Substituting olaparib, currently the only approved inhibitor in PDAC, with talazoparib in our PAD regimen enhanced its efficacy while maintaining comparable tolerability in vivo. Importantly, we show that PAD is an effective therapeutic regimen that can be extended to the most prevalent HR‐defective genotypes in PDAC including *ATM*, *BRCA1*, *BRCA2* and *PALB2* in a preclinical setting. Collectively, these data provide a strong rationale to implement the refined regimen, talazoparib‐based PAD, as a therapeutic concept tailored for HRD PDAC patients.

## Graphical abstract

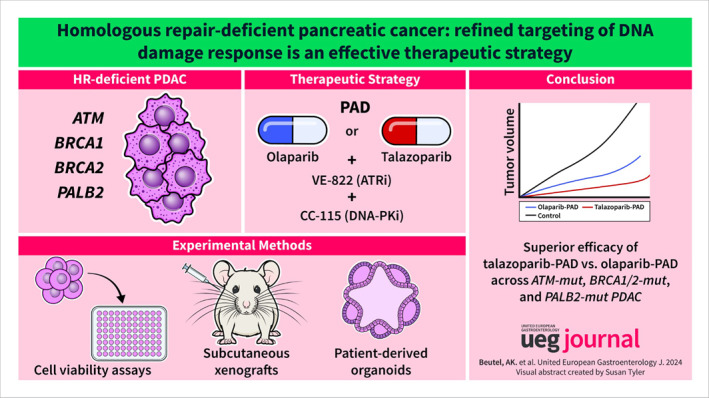

1

**Summary**

- Summarize the established knowledge on this subject
  ◦ Up to 20% of PDAC patients harbor somatic or germline mutations in HR‐related genes, but the PARP inhibitor (PARPi) olaparib is currently only approved for the small subset of g*BRCA1/2* mutation carriers.
  ◦ The value of PARPi and PARPi‐based treatments in other HRD genotypes has not been conclusively determined.
  ◦ Identifying the most effective PARPi to use in combination regimens may improve treatment strategies for HRD PDAC.
  ◦ The triple DNA repair inhibition strategy using the PARPi olaparib, ATR inhibitor VE‐822, and DNA‐PK inhibitor CC‐115 (termed PAD) is synthetic lethal in preclinical models of ATM deficiency.
- What are the significant and/or new findings of this study?
  ◦ In preclinical evaluation, talazoparib proves more potent than olaparib, the sole approved PARPi in PDAC, encouraging clinical repurposing of talazoparib‐based treatments.
  ◦ Talazoparib enhances efficacy of our previously reported multi‐drug DNA damage repair interference strategy (PAD) while maintaining comparable tolerability in vivo.
  ◦ Talazoparib‐based PAD is an optimized treatment that can be extended to the most prevalent HRD genotypes in PDAC including *ATM*, *BRCA1/2*, and *PALB2*, warranting clinical evaluation as a therapeutic concept tailored for HR‐deficient PDAC.

## 2 Introduction

Pancreatic ductal adenocarcinoma (PDAC) is a devastating malignancy presently ranking as the third leading cause of cancer‐related deaths in the United States [1]. PDAC features a complex mutational landscape, high inter‐ and intratumoral heterogeneity, and a remarkable therapy resistance, rendering its treatment notoriously difficult [2]. A biologically distinct subgroup of up to 20% of PDAC patients harbors somatic or germline mutations in *ATM*, *BRCA1/2*, *CHEK2*, *ATR*, *FANC*, *PALB2*, and other genes involved in homologous recombination (HR) repair [3], a crucial molecular mechanism for preserving genomic integrity. Among these, *BRCA1/2* and *PALB2* are recognized as core genes implicated in homologous recombination deficiency (HRD), a phenotype characterized by the incapacity to accurately repair DNA double‐strand breaks (DSBs) [4, 5]. Other non‐core mutations are suggested to induce HRD, but a consensus definition does not exist [6]. HRD tumors, which largely overlap with the unstable molecular subtype of PDAC [7], confer an increased sensitivity toward DNA damaging agents such as platinum derivates [7, 8, 9], DNA topoisomerase 1/2 inhibitors (TOPi) [10], and poly (ADP‐ribose) polymerase inhibitors (PARPi) [11, 12].

The PARPi olaparib has been approved for maintenance therapy in platinum sensitive germline (g) *BRCA1/2*‐mutated metastatic PDAC based on the phase 3 POLO trial [13, 14]. A recent phase 2 study revealed efficacy of rucaparib maintenance in PDAC patients with germline or somatic *BRCA1/2* or *PALB2* mutations, suggesting a potential benefit for PARPi beyond g*BRCA1/2* mutations [15]. Despite these advancements, there is an ongoing need to define which HRD genotypes are susceptible to PARPi treatment and to determine the optimal inhibitor. Clinically available PARPi vary widely in antitumor activity, primarily due to differences in their PARP trapping potency [16, 17]. Indeed, PARPi cytotoxicity is largely driven by its ability to trap PARP enzymes at DNA single‐strand breaks (SSBs) [16, 17], which provokes DSBs and forces HR deficient cells to rely on the error‐prone non‐homologous end joining (NHEJ) pathway for DSB repair. Among available PARPi, talazoparib is the structurally largest and most rigid inhibitor that traps PARP‐DNA complexes approximately 100‐fold more effectively than olaparib [17], the only inhibitor currently approved in PDAC.

However, primary and secondary acquired resistance to PARPi monotherapy is a major clinical challenge [18]. Therefore, simultaneous targeting of compensatory pathways within the DNA damage repair machinery, such as inhibition of ataxia telangiectasia and Rad3‐related (ATR) kinase or DNA‐dependent protein kinase (DNA‐PK), has emerged as a promising strategy for enhancing PARPi efficacy and circumventing resistance [19, 20]. Recently, we demonstrated synthetic lethality for the triple combination of the PARPi olaparib, ATR inhibitor VE‐822, and DNA‐PK inhibitor CC‐115 (termed PAD) in ATM‐deficient mouse and preclinical human models of PDAC [21].

In the present study, we systematically screened commercially available PARPi to determine their single agent potency to enhance the efficacy of our PAD regimen. Additionally, to broaden the eligible patient population, we systematically investigated the feasibility of expanding PARPi‐based treatments to prevalent HRD genotypes in PDAC. We identified talazoparib as the most promising single agent for our PAD regimen and observed a therapeutic advantage of employing talazoparib‐based PAD (PAD_tal_) instead of olaparib‐based PAD (PAD_ola_). Importantly, we demonstrated a HRD genotype‐specific effectiveness of the PAD regimen across *ATM*, *BRCA1*, *BRCA2* and *PALB2*‐defective versus HR‐proficient murine PDAC cells in vitro and in vivo. The efficacy of PAD_tal_ was confirmed in PDAC patient‐derived organoids, both with and without deleterious alterations in HR‐related genes. Collectively, these data provide a rationale to deploy the optimized regimen PAD_tal_ as a therapeutic concept tailored for HRD pancreatic cancer.

## 3 Results

### 3.1 Refining DNA Damage Repair Interference Strategy in ATM‐Deficient Pancreatic Cancer

BRCA‐deficient cells have been reported to be susceptible to treatment with DNA damage repair interfering agents such as platinum agents, TOPi, and PARPi, which differ in chemical, pharmacokinetic, and toxicological properties [7, 8, 10, 11, 12]. Accordingly, a careful selection of DNA‐damaging compounds is anticipated to substantially impact the therapeutic response of HRD tumors.

**FIGURE 1:**
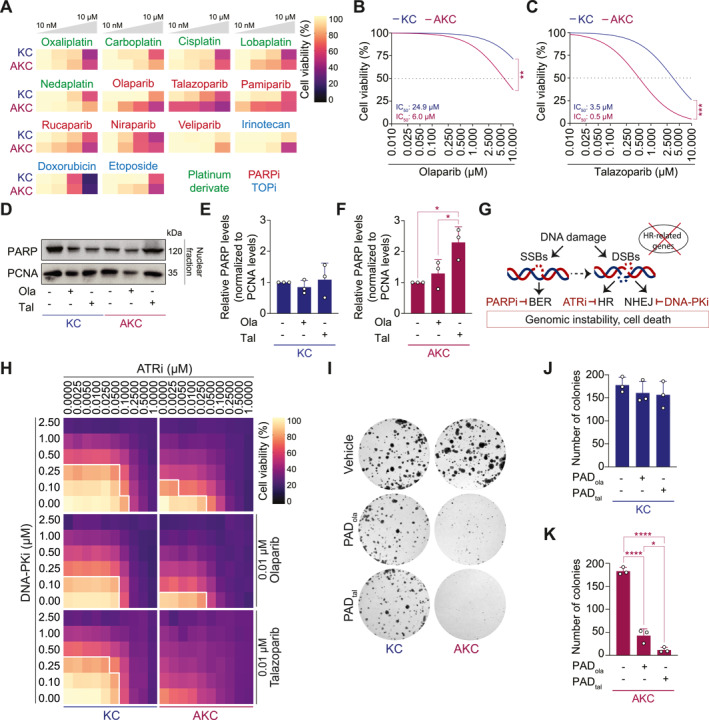
DNA damage response interference shows efficacy in murine ATM‐deficient pancreatic cancer cells. (A), Customized drug viability assay screen on *Kras* ^*LSL‐G12D/+*^; *Ptf1a* ^*Cre/+*^ (KC) and *Atm* ^*fl/fl*^; *Kras* ^*LSL‐G12D/+*^; *Ptf1a* ^*Cre/+*^ (AKC) cells with increasing doses (10 nM–10 μM) of PARP inhibitor (PARPi), platinum derivate, and topoisomerase inhibitor (TOPi) treatment. (B, C), Drug viability assay of olaparib (B) and talazoparib (C) treatment in KC and AKC cells. (D), Representative immunoblot of PARP trapping in nuclear fractions of KC and AKC cells after 4‐h treatment with vehicle, olaparib (10 μM) or talazoparib (10 μM). (E, F), Quantification of relative PARP levels normalized to PCNA levels in KC (E) and AKC (F) cells. Data are represented as mean ± SD. (G), Schematic representation of PAD (PARPi, ATR inhibitor (ATRi) and DNA‐PK inhibitor (DNA‐PKi)) triple therapy. (H), Drug viability assay on KC and AKC cells treated with varying concentrations of DNA‐PKi (CC‐115, 0.1–2.5 μM) and ATRi (VE‐822, 0.0025–1.0000 μM), plus 0.01 μM olaparib or talazoparib, respectively. Solid white lines delimit the area with a cell viability below 70%. (I–K), Representative images of colony formation assay (I) and quantification of colonies for vehicle, PAD_ola_ (DNA‐PKi CC‐115, 50 nM; ATRi VE‐822, 10 nM; and PARPi olaparib, 1 nM) and PAD_tal_ (DNA‐PKi CC‐115, 50 nM; ATRi VE‐822, 10 nM; and PARPi talazoparib, 1 nM) treatment in KC (J) and AKC (K) cells. Data are represented as mean ± SD. *, *p* ≤ 0.05; **, *p* ≤ 0.01; ***, *p* ≤ 0.001; ****, *p* ≤ 0.0001 by two‐tailed Student's t‐test (B, C) and one‐way ANOVA with Tukey post hoc test (E, F, J and K); KC versus AKC *p* = 0.0093 (B); KC versus AKC *p* = 0.0009 (C); vehicle versus Tal *p* = 0.0153, Ola versus Tal *p* = 0.0448 (F); vehicle versus PAD_ola_ *p* < 0.0001, vehicle versus PAD_tal_ *p* < 0.0001, PAD_ola_ versus PAD_tal_ *p* = 0.0219 (K). Ola, olaparib; Tal, talazoparib; SSB, DNA single‐strand break; DSB, DNA double‐strand break; BER, base excision repair; HR, homologous recombination; NHEJ: non‐homologous end joining.

To identify the most potent and genotype‐specific platinum derivate, TOPi, or PARPi in the context of ATM deficiency, we conducted a representative screening using ATM‐deficient (AKC, *Atm* ^*fl/fl*^; *Kras* ^*LSL‐G12D/+*^; *Ptf1a* ^*Cre/+*^) and ATM‐proficient (KC, *Kras* ^*LSL‐G12D/+*^; *Ptf1a* ^*Cre/+*^) primary murine PDAC cell lines (Figure 1A). Most platinum compounds as well as the TOP2i doxorubicin did not promote higher cytotoxic effects in ATM‐deficient cells compared to ATM‐proficient counterparts, in line with previous findings [21]. Conversely, the TOP2i etoposide and the TOP1i irinotecan were more effective in ATM‐null cells, albeit not very potent (Figure 1A). Intriguingly, tested PARPi demonstrated synthetic lethality with variable potencies in ATM‐deficient cells (Figure 1A). In AKC cells, olaparib, rucaparib, and pamiparib showed substantial cytotoxic effects (cell viability < 60%) at micromolar doses, while talazoparib caused comparable growth inhibitory effects already at nanomolar doses. In contrast, veliparib was least effective in our experimental setting, and niraparib showed overlapping potency in KC cells suggesting a reduced HRD specificity for this compound (Figure 1A). Determination of the half maximal inhibitory concentration (IC_50_) confirmed the genotype‐specific efficacy for olaparib and talazoparib in AKC compared to KC cells (Figure 1B,C). Importantly, talazoparib exhibited an overall more robust cytotoxic activity as reflected by lower IC_50_ values (Figure 1B,C). PARPi cytotoxicity is primarily driven by its ability to trap PARP enzymes at damaged DNA [16, 17, 22, 23]. Thus, we assessed the PARP trapping potency in nuclear fractions of KC and AKC cell lysates treated with olaparib or talazoparib (Figure 1D). Our results showed relevant PARP trapping only in AKC cells, supporting the observation that HR‐deficient cells are more vulnerable to PARP inhibition than their HR‐proficient counterparts (Figure 1D–F). Furthermore, talazoparib induced more pronounced PARP trapping compared to olaparib in AKC cells (Figure 1D,F). This is consistent with previous reports identifying talazoparib as the most potent PARP trapper, exhibiting the highest single‐agent activity among PARPi [16, 17, 22, 23].

Altogether, this nominates talazoparib as the most promising candidate in our inhibitor panel to effectively target ATM‐deficient malignant cells.

Single‐agent inhibition of DNA‐PK and ATR is known to disrupt major DSB repair pathways (Figure 1G) [24] and exerted cytotoxic effects in AKC cells (Supplementary Figure S1A,B). Accordingly, we have previously demonstrated a synergistic efficacy of simultaneous targeting of compensatory DNA damage repair pathways in the context of ATM deficiency [21, 25]. Our previous combination used the PARPi olaparib, ATRi VE‐822, and DNA‐PKi CC‐115 (PAD_ola_) [21, 25]. Given the higher efficacy achieved by talazoparib monotherapy (Figure 1A,C), we postulated that substituting olaparib with talazoparib in our PAD regimen would further enhance its potency. Hence, talazoparib was combined with ATRi VE‐822 and DNA‐PKi CC‐115 (PAD_tal_). To directly compare PAD_ola_ and PAD_tal_, we assessed cell viability by titrating ATRi and DNA‐PKi against different doses of olaparib and talazoparib (Figure 1H; Supplementary Figure S1C). While synthetic lethality in ATM‐deficient malignant cells was achieved with both PARPi, PAD_tal_ was markedly more potent than PAD_ola_ (Figure 1H; Supplementary Figure S1C). Clonogenicity assays confirmed the genotype‐specific effect of both PAD regimens in ATM‐deficient PDAC cells and highlighted the superior cytotoxicity of PAD_tal_ (Figure 1I–K).

### 3.2 DNA Damage Response Interference Is Effective Across Multiple HRD Genotypes

**FIGURE 2:**
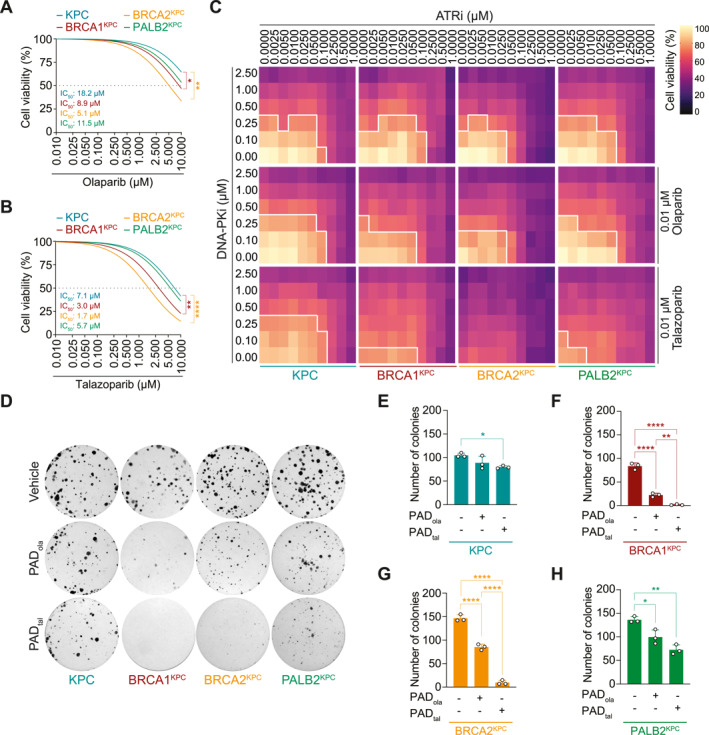
DNA damage response interference is efficacious across multiple homologous recombination‐deficient genotypes. (A, B), Drug viability assay of olaparib (A) and talazoparib (B) treatment in *Kras* ^*LSL‐G12D/+*^; *Trp53* ^*LSL‐R270H/+*^; *Pdx1*‐*Cre* (KPC), *Brca1* ^*fl/fl*^; *Kras* ^*LSL‐G12D/+*^; *Trp53* ^*LSL‐R270H/+*^; *Pdx1*‐*Cre* (BRCA1^KPC^), *Brca2* ^*fl/fl*^; *Kras* ^*LSL‐G12D/+*^; *Trp53* ^*LSL‐R270H/+*^; *Pdx1*‐*Cre* (BRCA2^KPC^), and *Palb2* ^*fl/fl*^; *Kras* ^*LSL‐G12D/+*^; *Trp53* ^*LSL‐R270H/+*^; *Pdx1*‐*Cre* (PALB2^KPC^) cells. (C), Drug viability assay on KPC, BRCA1^KPC^, BRCA2^KPC^, and PALB2^KPC^ cells treated with varying concentrations of DNA‐PK inhibitor (DNA‐PKi CC‐115, 0.1–2.5 μM) and ATR inhibitor (ATRi VE‐822, 0.0025–1.0000 μM), plus 0.01 μM olaparib or talazoparib, respectively. Solid white lines delimit the area with a cell viability below 70%. (D–H), Representative images of colony formation assay (D) and quantification of colonies for vehicle, PAD_ola_ (DNA‐PKi CC‐115, 50 nM; ATRi VE‐822, 10 nM; and PARPi olaparib, 1 nM) and PAD_tal_ (DNA‐PKi CC‐115, 50 nM; ATRi VE‐822, 10 nM; and PARPi talazoparib, 1 nM) treatment in KPC (E), BRCA1^KPC^ (F), BRCA2^KPC^ (G), and PALB2^KPC^ (H) cells. Data are represented as mean ± SD. *, *p* ≤ 0.05; **, *p* ≤ 0.01; ****, *p* ≤ 0.0001 by one‐way ANOVA with Tukey post hoc test (A and B, E–H). KPC versus BRCA1^KPC^ *p* = 0.0334, KPC versus BRCA2^KPC^ *p* = 0.0092 (A); KPC versus BRCA1^KPC^ *p* = 0.0079, KPC versus BRCA2^KPC^ *p* < 0.0001 (B); vehicle versus PAD_tal_ *p* = 0.0280 (E); vehicle vs. PAD_ola_ *p* < 0.0001, vehicle vs. PAD_tal_ *p* < 0.0001, PAD_ola_ vs. PAD_tal_ *p* = 0.0037 (F); vehicle versus PAD_ola_ *p* < 0.0001, vehicle versus PAD_tal_ *p* < 0.0001, PAD_ola_ versus PAD_tal_ *p* < 0.0001 (G); vehicle versus PAD_ola_ *p* = 0.0169, vehicle versus PAD_tal_ *p* = 0.0011 (H).

To validate the efficacy of therapeutic DNA damage response interference in a broader context that represents the spectrum of HR‐related mutations seen across PDAC patients, we expanded our experimental approach to *BRCA1*, *BRCA2*, and *PALB2* genotypes. Here, we assessed the cytotoxicity of olaparib and talazoparib in BRCA1‐deficient (BRCA1^KPC^, *Brca1* ^*fl/fl*^; *Kras* ^*LSL‐G12D/+*^; *Trp53* ^*LSL‐R270H/+*^; *Pdx1*‐*Cre*), BRCA2‐deficient (BRCA2^KPC^, *Brca2* ^*fl/fl*^; *Kras* ^*LSL‐G12D/+*^; *Trp53* ^*LSL‐R270H/+*^; *Pdx1*‐*Cre*), PALB2‐deficient (PALB2^KPC^, *Palb2* ^*fl/fl*^; *Kras* ^*LSL‐G12D/+*^; *Trp53* ^*LSL‐R270H/+*^; *Pdx1*‐*Cre*), and KPC (*Kras* ^*LSL‐G12D/+*^; *Trp53* ^*LSL‐R270H/+*^; *Pdx1*‐*Cre*) primary murine PDAC cell lines. In accordance with our results in ATM‐null cells (Figure 1B,C), depletion of BRCA1, BRCA2, or PALB2 sensitized KPC cells toward PARP inhibition (Figure 2A,B). While HRD genotypes showed susceptibility to both PARPi, talazoparib was again more potent than olaparib as illustrated by lower IC_50_ values (Figure 2A,B). Single‐agent DNA‐PK and ATR inhibition exhibited cytotoxic effects in HRD cells (Supplementary Figure S1D,E), and their combination with olaparib (PAD_ola_) proved to be more effective in BRCA1, BRCA2, and PALB2‐deficient cells as compared to their HR‐proficient KPC control (Figure 2C; Supplementary Figure S1F). Again, PAD_tal_ therapy was substantially more efficient than PAD_ola_ in killing BRCA1/2 and PALB2‐null cells, consistent with our observations employing ATM‐null cells (Figure 1H; Supplementary Figure S1C). Colony formation assays substantiated that particularly PAD_tal_ exhibited higher anticancer activity against BRCA1/2^KPC^ and PALB2^KPC^ cells as compared to KPC counterparts (Figure 2D–H). Collectively, these findings demonstrate the feasibility of extending the PAD regimen to other frequent HRD genotypes, and an advantage of PAD_tal_ over PAD_ola_ in eradicating ATM, BRCA1/2, and PALB2‐deficient cells.

### 3.3 Talazoparib‐Based PAD Treatment Achieves Tumor Control in ATM‐Deficient PDAC

**FIGURE 3:**
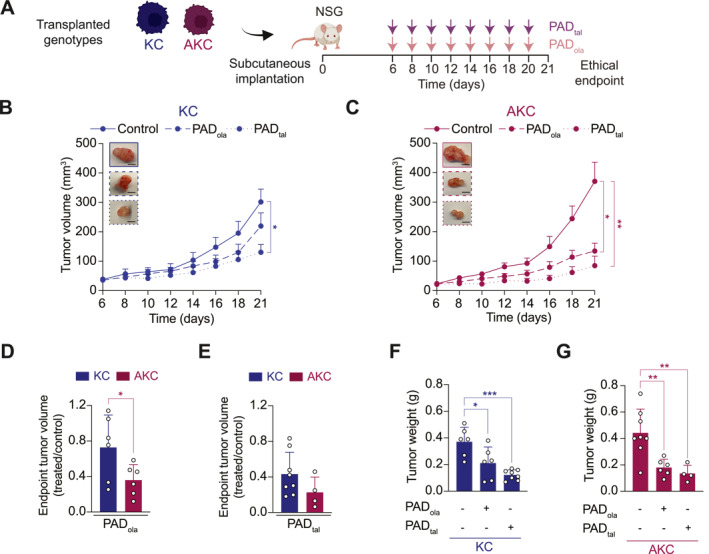
Talazoparib‐based PAD decreases ATM‐deficient tumor growth in vivo. (A), Schematic representation of the subcutaneous assay shown in (B, C) with treatment administration schedule. (B, C), Time‐dependent development of tumor volumes arising from *Kras* ^*LSL‐G12D/+*^; *Ptf1a* ^*Cre/+*^ (KC) (B) and *Atm* ^*fl/fl*^; *Kras* ^*LSL‐G12D/+*^; *Ptf1a* ^*Cre/+*^ (AKC) (C) cells treated with PAD_ola_ (ATR inhibitor VE‐822, 20.0 mg/kg; DNA‐PK inhibitor CC‐115, 2.5 mg/kg; and PARP inhibitor olaparib, 50.0 mg/kg), or PAD_tal_ (ATR inhibitor VE‐822, 20.0 mg/kg; DNA‐PK inhibitor CC‐115, 2.5 mg/kg; and PARP inhibitor talazoparib, 0.1 mg/kg), with representative macroscopic images of resected tumors. Data are represented as mean ± SEM. Scale bars represent 5 mm. (D, E), Final tumor volumes of KC and AKC allografts treated with PAD_ola_ (D) or PAD_tal_ (E). Data are represented as mean ± SD. (F, G), Quantification of tumor weight from KC (F) and AKC (G) allografts at endpoint. Data are represented as mean ± SD. *, *p* ≤ 0.05; ***, *p* ≤ 0.001 by one way ANOVA with Tukey post hoc test (B, C, F, G) and two‐tailed Student's *t*‐test (D, E). Control versus PAD_tal_ *p* = 0.0109 (B), control versus PAD_ola_ *p* = 0.0145, control versus PAD_tal_ *p* = 0.0096 (C); *p* = 0.0500 (D); control versus PAD_ola_ *p* = 0.0193, control versus PAD_tal_ *p* = 0.0003 (F); control versus PAD_ola_ *p* = 0.0057, control versus PAD_tal_ *p* = 0.0044 (G).

To determine the in vivo potency of the different PAD regimens in an ATM‐deficient context, AKC and KC allograft tumors were established and monitored for therapeutic responses (Figure 3A). As previously described by our group [21], PAD_ola_ treatment inhibited tumor growth more effectively in AKC allografts compared to KC allografts (Figure 3B–D). PAD_tal_ reduced tumor growth in KC and AKC allografts (Figure 3B,C,E) and led to lower tumor weights in both genotypes at endpoint (Figure 3F,G). Although performing less genotype‐specific, PAD_tal_ achieved a robust tumor control in AKC allografts, suggesting a therapeutic benefit over PAD_ola_ (Figure 3C). Both PAD regimens caused comparable body weight loss in NSG mice that remained within an acceptable range (Supplementary Figure S2A), in line with previous findings for PAD_ola_ therapy [21].

### 3.4 DNA Damage Repair Interference Suppresses Tumor Growth Across Multiple HRD PDAC Genotypes

**FIGURE 4:**
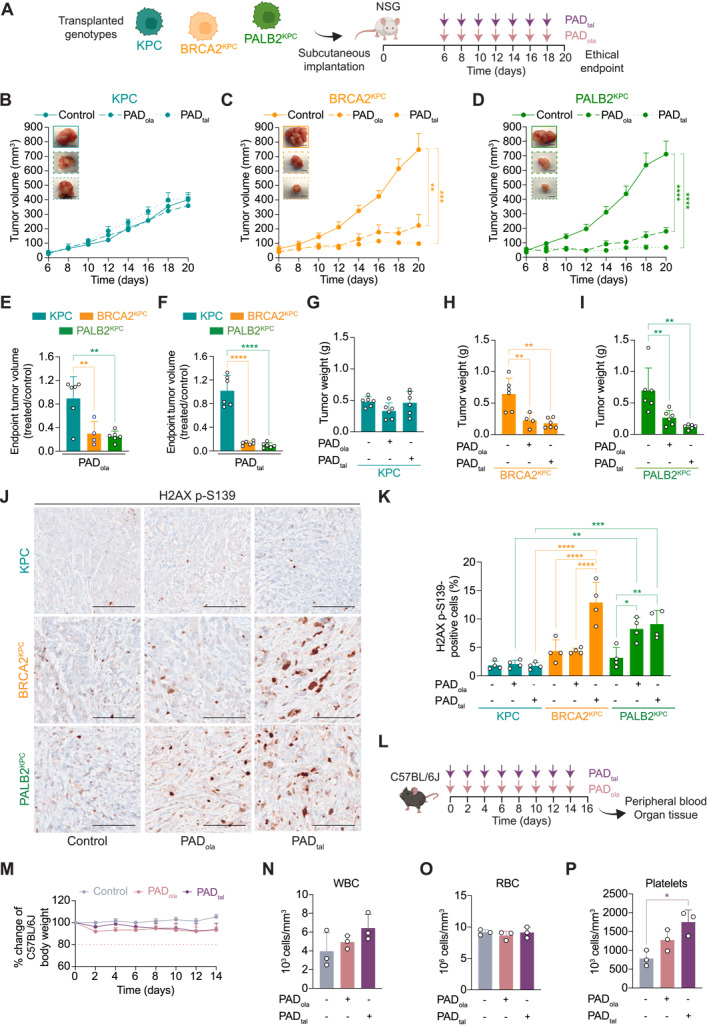
Talazoparib‐based PAD achieves tumor control in homologous recombination‐deficient pancreatic cancer. (A), Schematic representation of subcutaneous assay shown in (B–D) with treatment administration schedule. (B–D), Time‐dependent development of tumor volumes arising from *Kras* ^*LSL‐G12D/+*^; *Trp53* ^*LSL‐R270H/+*^; *Pdx1*‐*Cre* (KPC) (B), *Brca2* ^*fl/fl*^; *Kras* ^*LSL‐G12D/+*^; *Trp53* ^*LSL‐R270H/+*^; *Pdx1*‐*Cre* (BRCA2^KPC^) (C), *and Palb2* ^*fl/fl*^; *Kras* ^*LSL‐G12D/+*^; *Trp53* ^*LSL‐R270H/+*^; *Pdx1*‐*Cre* (PALB2^KPC^) (D) cells treated with PAD_ola_ (ATR inhibitor VE‐822, 20.0 mg/kg; DNA‐PK inhibitor CC‐115, 2.5 mg/kg; and PARP inhibitor olaparib, 50.0 mg/kg), or PAD_tal_ (ATR inhibitor VE‐822, 20.0 mg/kg; DNA‐PK inhibitor CC‐115, 2.5 mg/kg; and PARP inhibitor talazoparib, 0.1 mg/kg), with representative macroscopic images of resected tumors. Data are represented as mean ± SEM. Scale bars represent 5 mm. (E, F), Final tumor volumes of KPC, BRCA2^KPC^, and PALB2^KPC^ allografts treated with PAD_ola_ (E) and PAD_tal_ (F). Data are represented as mean ± SD. (G–I), Quantification of tumor weight from KPC (G), BRCA2^KPC^ (H) and PALB2^KPC^ (I) allografts at endpoint. Data are represented as mean ± SD. (J, K), Immunohistochemistry staining for H2AX p‐S139 (J) and quantification of H2AX p‐S139‐positive cells (K) in resected tumors. Scale bars represent 100 μm. Data are represented as mean ± SD. (L), Schematic representation of toxicity assay in C57B/6J mice with treatment administration schedule. (M), Percentage change of C57B/6J body weight under treatment with PAD_ola_, and PAD_tal_, respectively. The horizontal dotted line represents 20% weight loss. (N–P), Peripheral blood cell count of white blood cells (WBC) (N), red blood cells (RBC) (O) and platelets (P) in C57B/6J mice following treatment with PAD_ola_, and PAD_tal_, respectively. **, *p* ≤ 0.01; ***, *p* ≤ 0.001; ****, *p* ≤ 0.0001 by one‐way ANOVA with Tukey post hoc test (B–I, N–P) and two‐way ANOVA with Tukey post hoc test (K). Control versus PAD_ola_ *p* = 0.0019, control versus PAD_tal_ *p* = 0.0001 (C); control versus PAD_ola_ *p* < 0.0001, control versus PAD_tal_ *p* < 0.0001 (D); KPC versus BRCA2^KPC^ *p* = 0.0083, KPC versus PALB2^KPC^ *p* = 0.0021 (E); KPC versus BRCA2^KPC^ *p* < 0.0001, KPC versus PALB2^KPC^ *p* < 0.0001 (F); control versus PAD_ola_ *p* = 0.0066, control versus PAD_tal_ *p* = 0.0012 (H); control versus PAD_ola_ *p* = 0.0098, control versus PAD_tal_ *p* = 0.0010 (I); KPC PAD_ola_ versus PALB2^KPC^ PAD_ola_ *p* = 0.0034, KPC PAD_tal_ versus BRCA2^KPC^ PAD_tal_ *p* < 0.0001; KPC PAD_tal_ versus PALB2^KPC^ PAD_tal_ *p* = 0.0004; BRCA2^KPC^ control versus BRCA2^KPC^ PAD_tal_ *p* < 0.0001; BRCA2^KPC^ PAD_ola_ versus BRCA2^KPC^ PAD_tal_ *p* < 0.0001; PALB2^KPC^ control versus PALB2^KPC^ PAD_ola_ *p* = 0.0223; PALB2^KPC^ control versus PALB2^KPC^ PAD_tal_ *p* = 0.0051 (K); control versus PAD_tal_ *p* = 0.0118 (P).

The potential translation of our multi‐DNA damage repair interference concept to other prevalent HRD cancer genotypes was assessed in vivo using KPC, BRCA2^KPC^, and PALB2^KPC^ allograft tumor models (Figure 4A). In contrast to their respective HR‐proficient control genotype, BRCA2^KPC^ and PALB2^KPC^ tumor growth was markedly reduced upon PAD_ola_ treatment (Figure 4B–D). Strikingly, PAD_tal_ almost completely abolished tumor development of both BRCA2^KPC^ and PALB2^KPC^ over the course of treatment, while not affecting KPC tumor growth (Figure 4B–D). Both regimens performed in a genotype‐specific fashion in HRD (Figure 4E,F) and led to smaller tumors in BRCA2^KPC^ and PALB2^KPC^ allografts at endpoint (Figure 4G–I). Phosphorylation of H2AX at serine 139 is a sensitive early indicator of DNA damage. Therefore, we assessed levels of H2AX p‐S139 by immunohistochemistry in resected KPC, BRCA2^KPC^, and PALB2^KPC^ allografts (Figure 4J). Here, we observed a concomitant induction of DNA damage in BRCA2^KPC^ and PALB2^KPC^ tumors upon PAD treatments, in contrast to KPC tumor tissues (Figure 4J,K). The extent of mouse body weight loss remained within acceptable limits for both PAD therapies (Supplementary Figure S2B), consistent with our observations in KC and AKC tumor‐bearing mice (Supplementary Figure S2A). Altogether, this demonstrates the potential of our combinational therapy PAD_tal_ and its therapeutic advantage over PAD_ola_ in suppressing BRCA2 and PALB2‐deficient tumor growth in vivo.

To further assess potential adverse effects of our proposed triple therapy regimens, we conducted a toxicity assay in an immunocompetent mouse model using C57BL/6J mice (Figure 4L). Fourteen days of treatment with PAD_ola_ and PAD_tal_, respectively, resulted in only minor body weight loss (Figure 4M). Notably, no difference in weight loss was observed between the two regimens. Peripheral bloods counts showed no evidence of leukopenia, anemia, or thrombocytopenia in either treatment condition (Figure 4N–P). Resected pancreas, liver, and kidney tissues did not display relevant levels of DNA damage, as assessed by immunohistochemistry for H2AX p‐S139 (Supplementary Figure S2C). In the intestine, minor H2AX p‐S139 positivity was observed in the proliferative stem cells residing in the crypt of the intestinal glands, but no relevant difference was found between control and either treatment condition (Supplementary Figure S2C,E). Immunohistochemistry stainings for cleaved caspase‐3 (CC3), used as a programmed cell death marker, did not reveal apoptotic cells in any of the organ tissues (Supplementary Figure S2D). Altogether, no evidence of severe acute toxicity was observed from PAD treatments.

### 3.5 Talazoparib‐Based PAD Is Effective in HRD PDAC Patient‐Derived Organoids

**FIGURE 5:**
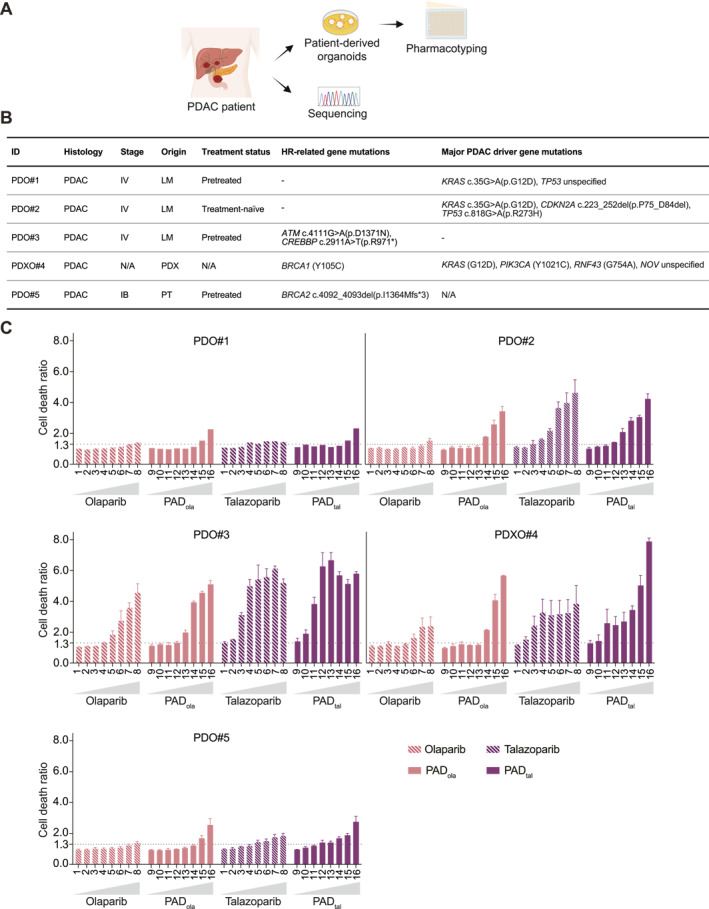
Talazoparib‐based PAD is an effective regimen in homologous recombination repair‐deficient PDAC patient‐derived organoids. (A), Schematic representation of patient‐derived organoids (PDOs) used for pharmacotyping. (B), Table depicting basic clinical information and major oncogenic mutations of patients' tumor tissue. (C), Cell death ratio (CDR) using PDOs treated with PAPRi olaparib or talazoparib, respectively (concentrations: 1, 0.082 μM; 2, 0.205 μM; 3, 0.512 μM; 4, 1.28 μM; 5, 3.2 μM; 6, 8 μM; 7, 20 μM; 8, 50 μM); PAD_ola_ or PAD_tal_, respectively (concentrations: 9, PARPi 0.082 μM, DNA‐PKi 4.096 nM and ATRi 2.048 nM; 10, PARPi 0.205 μM, DNA‐PKi 10.24 nM and ATRi 5.12; 11, PARPi 0.512 μM, DNA‐PKi 25.6 nM and ATRi 12.8 nM; 12, PARPi 1.28 μM, DNA‐PKi 64 nM and ATRi 32 nM; 13, PARPi 3.2 μM, DNA‐PKi 0.16 μM and ATRi 80 nM; 14, PARPi 8 μM, DNA‐PKi 0.4 μM and ATRi 0.2 μM; 15, PARPi 20 μM, DNA‐PKi 1 μM and ATRi 0.5 μM; 16, PARPi 50 μM, DNA‐PKi 2.5 μM and ATRi 1.25 μM). PDOs are considered sensitive when CDR > 1.3 [21]. LM, liver metastasis; N/A, not available; PDAC, pancreatic ductal adenocarcinoma; PDO, patient‐derived organoid; PDX, patient‐derived xenograft; PDXO, patient‐derived xenograft‐derived organoid; PT, primary tumor.

Utilization of genetically defined murine cells minimizes variability among different genotypes but fails to adequately capture patient heterogeneity. Patient‐derived organoids (PDOs) recapitulate morphological, genetic, and epigenetic characteristics of the original tumor and reflect intra‐tumoral heterogeneity [26, 27, 28]. Therefore, we validated the efficacy of our proposed therapies in a set of five organoid lines (four PDO lines and one patient‐derived xenograft‐derived organoid (PDXO) line) isolated from PDAC patients with or without deleterious alterations in HR‐related genes (Figure 5A). Organoid lines were selected based on results from gene panel sequencing conducted on patient tumor material or blood sample, irrespective of cancer stage, tumor tissue origin, or treatment status. Pathogenic class IV/V mutations in HR‐related genes and major PDAC driver genes are depicted in Figure 5B. We included two PDO lines derived from HRP patient tumors (PDO#1 and PDO#2) that were randomly selected from the PDAC organoid library of the Organoid Core Facility of the Medical Faculty at Ulm University, Germany. Three PDO lines were derived from PDACs harboring class IV/V mutations in HR‐related genes, including *ATM* (PDO#3), *BRCA1* (PDXO#4), and *BRCA2* (PDO#5) (Figure 5B). We tested a broad range of concentrations for single‐agent olaparib and talazoparib, as well as for the combination treatments PAD_ola_ and PAD_tal_. Cell death ratio (CDR) was assessed as a readout for cytotoxicity and PDO lines were considered sensitive at CDR values above 1.3, as previously described [21, 29]. HRP PDO#1 was overall least responsive to tested treatments, while HRP PDO#2 showed positive CDRs at higher concentrations, reflective of patient heterogeneity between HRP lines. Interestingly, *ATM*‐mutated PDO#3 and *BRCA1*‐mutated PDO#4 were most susceptible to PAD_tal_ (Figure 5C). Strikingly, PAD_tal_ caused cell death at all tested concentrations in PDO#3 and at seven out of eight concentrations in PDO#4 (Figure 5C). The *BRCA2*‐mutated line (PDO#5) showed no relevant cytotoxicity to olaparib monotherapy with CDR values below 1.3, suggestive of primary resistance [18]. Intriguingly, talazoparib was still effective at the highest four concentrations and PAD_tal_ caused relevant cell death across five concentrations, suggesting that PAD_tal_ may be a potent regimen even in olaparib refractory situations (Figure 5C). Altogether, our data highlight PAD_tal_ as an effective DNA‐damaging regimen to target HRD PDOs.

**FIGURE 6:**
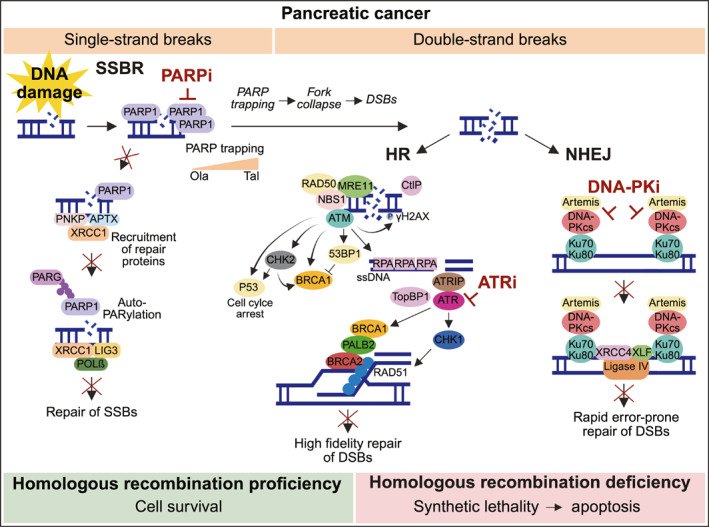
PAD triple regimen inhibits compensatory DNA damage repair pathways. Schematic representation of compensatory DNA damage repair pathways targeted by PARP inhibitors, ATR inhibitors and DNA‐PK inhibitors. ATRi, ATR inhibitor; DNA‐PKi, DNA‐PK inhibitor; DSB, double‐strand break; HR, homologous recombination; NHEJ, non‐homologous‐end joining; PARPi, PARP inhibitor; ssDNA, single strand DNA; SSBR, single‐strand break repair. Image created with BioRender.com.

The principle of our proposed PAD triple therapy in HRD and the DNA repair pathways targeted by PARPi, DNA‐PKi and ATRi are summarized in Figure 6. Conceptually, the formation of toxic PARP‐DNA complexes and accumulation of unrepaired SSBs provoke DSBs, which cannot be repaired efficiently in HRD cells. Talazoparib, the most potent PARP trapping agent [16, 17, 22, 23], induces superior cytotoxicity than olaparib. Simultaneous inhibition of ATR, a kinase orchestrating multifaceted responses to DNA replication stress to maintain genome integrity, potentiates PARPi cytotoxicity and overcomes PARPi resistance in HRD [19, 30]. Simultaneous targeting of DNA‐PK, a kinase involved in the more error‐prone NHEJ repair pathway of DSBs, further enhances PARPi efficacy [20, 31]. Thus, concurrent targeting of compensatory pathways within the DDR machinery leads to augmented genomic instability and apoptosis in HRD (Figure 6).

## 4 Discussion

Considering the modest survival benefit of conventional chemotherapy for advanced PDAC patients, the emergence of personalized treatment approaches brings new hope. Clinical guidelines recommend routine testing for HR‐related mutations in newly diagnosed advanced PDAC patients [32], paving the way for therapeutically exploiting HRD‐caused vulnerabilities in clinical care. The PARPi olaparib has recently been incorporated into the treatment paradigm for PDAC patients [13, 14]. However, it confers benefits to merely 4.6% of g*BRCA1/2* mutation carriers [33], and its applicability is contingent upon prior response to platinum‐based therapies, which substantially narrows the eligible patient population. Despite improvements in progression‐free survival, the lack of overall survival benefit and the variable clinical responses to PARPi remain a major challenge. Only a few patients with g*BRCA* mutations are long‐term responders. Most patients have durable responses followed by acquired resistance, and some patients experience primary resistance [18]. Thus, the inevitable acquisition of resistance to olaparib monotherapy underlines the necessity to optimize PARPi strategies in PDAC. This includes identifying the optimal inhibitor and developing effective combination therapies. Furthermore, there is an urgent need to characterize HRD‐causing genotypes that are susceptible to PARP inhibition beyond g*BRCA1/2* to expand the eligible patient population. In the present study, we aimed to (i) identify the most potent DNA damage repair interference agent to enhance the efficacy of our previously reported multi‐DNA repair inhibition treatment [21] and (ii) systematically investigate the effectiveness of PARPi‐based treatments within defined HRD genotypes.

As a first step, we aimed to identify the most potent inhibitor in the context of ATM deficiency among platinum derivates, TOPi, and PARPi, all of which have been reported to be effective in *BRCA* mutations [7, 8, 10, 11, 12]. In our customized screen, most platinum derivates and the TOP2i doxorubicin did not exhibit genotype‐specific activity in ATM deficiency. We observed a more consistent synthetic lethal interaction across tested PARPi. Talazoparib, the largest and most rigid inhibitor among PARPi [17], featured more pronounced PARP trapping than olaparib in AKC cells. These findings align with previous reports showing that talazoparib traps PARP‐DNA complexes more effectively than olaparib and displays higher cytotoxicity [16, 17, 22, 23, 34, 35]. Indeed, single‐agent talazoparib exhibited a greater potency than olaparib across ATM, BRCA1/2, and PALB2‐deficient cells. Supporting our finding, a recent phase 2 clinical trial demonstrated efficacy of talazoparib monotherapy in pretreated patients with solid tumors and mutations in HR pathway genes other than *BRCA1/2* [36]. Remarkably, all patients harboring a *gPALB2* mutation (*n* = 5 breast cancer patients and *n* = 1 PDAC patient) experienced a noticeable tumor shrinkage following the therapy [36]. Veliparib, the PARPi with the lowest PARP trapping potency, was least efficient in our experimental setting, in line with phase 2 trials reporting a lack of clinical activity of this compound in g*BRCA* or g*PALB2*‐mutated PDAC [37, 38].

Recognizing the remarkable potency of single‐agent talazoparib and preemptively addressing acquisition of resistance to PARPi monotherapy, we replaced olaparib with talazoparib in our previously reported PAD combination therapy [21]. PAD treatments exhibited a robust growth inhibitory effect on ATM, BRCA2, and PALB2‐deficient allografts in vivo, indicating the feasibility of extending our treatment combination across multiple HRD genotypes in PDAC. In terms of efficacy, PAD_tal_ was more favorable than PAD_ola_ while maintaining comparable tolerability in vivo.

Although these safety results are encouraging, conclusions from short‐term rodent toxicity assays cannot be applied to predict long‐term treatments in patients. So far, clinical studies have indicated tolerability of these drugs as monotherapy or dual combination. Talazoparib has been approved for patients with g*BRCA*‐mutated, HER2‐negative breast cancer based on a phase 3 study that reported acceptable adverse events [39]. Similarly, a phase 1 study in advanced solid tumors found tolerability of a monotherapy with DNA‐PKi [40]. A combination therapy of PARPi and ATRi in a phase 2 trial in ovarian cancer [41], as well as preliminary results from a phase 1 dose‐escalation trial in various cancers including PDAC [42] demonstrated manageable safety in patients.

Simultaneous targeting of compensatory pathways within the DNA damage repair machinery offers the advantages of preventing and overcoming resistance and reducing single‐agent concentrations. However, as a triple drug combination, a major challenge for translating PAD into clinical trials will be addressing combinatorial toxicity concerns, in particular myelosuppression and gastrointestinal adverse events. Ultimately, early clinical trials will be crucial to evaluate patient safety.

To substantiate our findings in human model systems that reflect patient heterogeneity, we employed PDOs. We validated the efficacy of our proposed therapies in five PD(X)O lines, two of which were derived from HR‐proficient PDAC tumors and three that harbored a pathogenic class IV/V mutation in *ATM*, *BRCA1*, or *BRCA2* respectively. Intriguingly, *ATM* and *BRCA1*‐mutated PD(X)Os were exquisitely vulnerable to single‐agent PARPi and combinational PAD treatments. Notably, PAD_tal_ induced the most pronounced cytotoxicity, indicating its efficacy in HRD PDOs. Interestingly, the *BRCA2*‐mutated PDO line was less affected by single‐agent olaparib potentially mirroring the clinical scenario of primary resistance [18]. PAD_tal_ restored therapy response of this PDO line, underpinning the potency of a multi‐drug DNA repair interference.

In summary, our data favors talazoparib over olaparib, the only approved PARPi in PDAC, encouraging clinical evaluation of talazoparib‐based therapies. Moreover, our preclinical work indicates a therapeutic advantage of PAD_tal_ compared to PAD_ola_ while maintaining similar tolerability in vivo. Importantly, we systematically demonstrate efficacy of our refined treatment combination PAD_tal_ across predominant HRD genotypes including *ATM*, *BRCA1*, *BRCA2*, and *PALB2*, in mouse and human model systems of PDAC. Thus, our work outlines a strong rationale to extend the indication of PARPi beyond the small subset of g*BRCA1/2* mutation carriers, to encompass up to 20% of PDAC patients. Collectively, these data propose PAD_tal_ as an optimized therapeutic strategy tailored specifically for HRD PDAC, warranting clinical investigation.

## 5 Material and Methods

### 5.1 Cell Culture

*Kras* ^*LSL‐G12D/+*^; *Ptf1a* ^*Cre/+*^ (KC) and *Atm* ^*fl/fl*^; *Kras* ^*LSL‐G12D/+*^; *Ptf1a* ^*Cre/+*^ (AKC) cells were isolated from *Kras* ^*LSL‐G12D/+*^; *Ptf1a* ^*Cre/+*^ and *Atm* ^*fl/fl*^; *Kras* ^*LSL‐G12D/+*^; *Ptf1a* ^*Cre/+*^ mice and immortalized as previously described [43]. *Brca1* ^*fl/fl*^; *Kras* ^*LSLG12D/+*^; *Trp53* ^*LSL‐R270H/+*^; *Pdx1*‐*Cre* (BRCA1^KPC^), *Brca2* ^*fl/fl*^; *Kras* ^*LSL‐G12D/+*^; *Trp53* ^*LSL‐R270H/+*^; *Pdx1*‐*Cre* (BRCA2^KPC^), *Palb2* ^*fl/fl*^; *Kras* ^*LSL‐G12D/+*^; *Trp53* ^*LSL‐R270H/+*^; *Pdx1*‐*Cre* (PALB2^KPC^), and *Kras* ^*LSL‐G12D/+*^; *Trp53* ^*LSL‐R270H/+*^; *Pdx1*‐*Cre* (KPC) were a kind gift from Dr. Dongju Park (Department of Cancer Biology and Genetics; Ohio State University; Columbus, OH, USA) [44]. Cells were cultured in DMEM (Thermo Fisher Scientific), containing 10% FBS (PAN Biotech) and 1% P/S (100 IU/mL penicillin and 100 μg/mL streptomycin sulfate; Thermo Fisher Scientific) and propagated at 37°C under 5% (v/v) CO_2_ atmosphere. Carboplatin (JM‐8), CC‐115, cisplatin, doxorubicin (adriamycin), etoposide, irinotecan (CPT‐11), lobaplatin (D‐19466), nedaplatin, niraparib (MK‐4827), olaparib (AZD2281), oxaliplatin (L‐OHP), pamiparib (BGB‐290), rucaparib (AG‐014699), talazoparib (BMN‐673), VE‐822 (berzosertib), and veliparib (ABT‐888) were purchased from Selleckchem.

### 5.2 Cell Viability Assays

Cells were seeded in 96‐well plates (2000 cells per well). Twenty‐four hours after seeding, cells were treated with cytotoxic agents for 3 days. Cell viability was analyzed by MTT assay (Sigma‐Aldrich) as described previously [45]. Absorbance was measured at 590 nm wavelength using a spectrophotometer (Tecan Infinite M200 Pro). Viability percentages were normalized to vehicle‐treated cell viability. Cell viability for IC_50_ determination and drug combination assays was assessed in 2 cell lines across 3 independent experiments.

### 5.3 Colony Formation Assays

Cells were seeded in triplicates in 6‐well plates (1000 cells per well) and treated 24 h after seeding. Following cultivation for seven to 10 days, cells were fixed with methanol and stained with 1% crystal violet solution (Sigma‐Aldrich). Images of the plates were imported into ImageJ software (National Institutes of Health), where a threshold was set to highlight the colonies. The binary watershed processing tool was applied to separate touching colonies. Colony size for counting was pre‐defined as greater than 0.1 mm^2^, and the counting was performed automatically.

### 5.4 Immunoblot for PARP Trapping

Three million cells were seeded into 10 cm dishes and treated after 24 h with 10 μM olaparib, talazoparib, or DMSO for 4 h. Whole cell lysates were collected, and nuclear fractions were isolated using the nuclear and cytoplasmic extraction reagents kit (Thermo Scientific). Lysates were quantified using Bradford protein assay (Roth). Protein amounts were equalized and run on 10% SDS gels, then transferred to PVDF transfer membranes (Millipore) and blocked with 5% milk‐TBST. After washing and incubation in the primary antibody anti‐PARP (Cell Signaling, 9542), or anti‐PCNA (Cell Signaling, 2586), membranes were washed and incubated in secondary antibodies. Membranes were then visualized on western blot and chemiluminescence imaging system (Fusion Solo X, Vilber) using chemiluminescent substrates (Supersignal West Dura, Thermo Fisher Scientific). Quantification of three independent experiments was performed using ImageJ software.

### 5.5 Subcutaneous Allografts

KC, AKC, BRCA2^KPC^, PALB2^KPC^, and KPC cells were implanted by subcutaneous injection of 8 × 10^5^ cells in 100 μL of 1:1 serum‐free DMEM:BME (Cultrex Reduced Growth Factor Basement Membrane Matrix, Type R1, Fisher) into the flanks of 10‐15‐week‐old female NOD‐*scid* IL2Rgamma^null^ (NSG) mice. For each treatment group, *n* ≥ 6 allografts were implanted. Mice were maintained in conventional health status‐controlled animal facilities with access to standard diet, dietary supplement (DietGel 76A, Clear H20) and water. Treatment was initiated on day 6 with PAD_ola_ (olaparib (50.0 mg/kg), VE‐822 (20.0 mg/kg), and CC‐115 (2.5 mg/kg) by i.p. injection every other day as described previously [21]) or PAD_tal_ (VE‐822 (20.0 mg/kg), and CC‐115 (2.5 mg/kg) by i.p. injection and talazoparib (0.1 mg/kg) by oral gavage every other day). Mice were euthanized on day 20 (BRCA2^KPC^, PALB2^KPC^, and KPC) and day 21 (KC, AKC), respectively, or when a predefined ethical endpoint was reached. Tumor size was measured with a caliper every 2 or 3 days. Tumor volumes were determined with the following equation: *v* = (*l* × *w* ^2^) × π/6 (*v* is volume, *l* is length, and *w* is width). After euthanization, subcutaneous tumors were resected, fixed in 4% formaldehyde at 4°C for 24 h and embedded in paraffin for histological analysis.

### 5.6 Toxicity Assay

Eight‐week‐old male C57BL/6J mice were treated with PAD_ola_ olaparib (50.0 mg/kg), VE‐822 (20.0 mg/kg), and CC‐115 (2.5 mg/kg) by i.p. injection every other day as described previously [21]) or PAD_tal_ (VE‐822 (20.0 mg/kg), and CC‐115 (2.5 mg/kg) by i.p. injection and talazoparib (0.1 mg/kg) by oral gavage every other day). *N* = 3 mice were used for treatment and control groups. Mice were maintained in conventional health status‐controlled animal facilities with access to standard diet, dietary supplement (DietGel 76A, Clear H20) and water. Mice were euthanized on day 16. Peripheral blood was collected by cardiac puncture into K2EDTA collection tubes (Microtainer, BD). Peripheral blood counts were analyzed with a scil vet ABC hematology analyzer (Scil animal care company). Resected liver, kidney, pancreas and intestines were fixed in Z‐fix solution (Anatech) and embedded in paraffin for histology.

### 5.7 Immunohistochemistry

Resected paraffin‐embedded tumors were sectioned to obtain 4 μm thick sections and stained for histological analysis. Rabbit monoclonal antibody against H2AX p‐S139 (1:250, clone 20E3, Cell Signaling) was used as a sensitive marker for DNA damage in subcutaneous allograft assays. Rabbit monoclonal antibodies against H2AX p‐S139 (1:1,000, Cell Signaling) and cleaved caspase‐3 (Asp175) (5A1E) (1:1,000, Cell Signaling) were used to stain organ tissues for the toxicity assay. H2AX p‐S139‐positive cells of bright‐field images were quantified using quPath v0.5.1 (https://qupath.github.io/), an open‐source software for digital pathology image analysis [46]. A second independent investigator who was blinded to the identity of the images performed the quantification via quPath. Images were imported and cell‐based detection parameters were manually set and optimized. Quantification was performed identically for all images stained with the same antibody. H2AX pS139‐positive cells or cleaved caspase‐3‐positive cells, respectively, were counted automatically in positive cell detection mode using optical density sum. Results were quality controlled by visual inspection. All quantifications were performed on five arbitrary images of four subcutaneous tumors, or on five arbitrary images of organ tissues from three mice included in the toxicity assay.

### 5.8 Isolation and Culture of Patient‐Derived Organoids

Organoids were isolated as previously described [47, 48, 49], from ultrasound‐guided biopsies of liver metastases, surgical resection of the primary tumor, or PDX [50]. Cells were cultured in 50 μL domes of BME (Cultrex Reduced Growth Factor Basement Membrane Extract, Type 2, Pathclear, R&D Systems) and covered with organoid growth medium, either human complete medium (hCPLT, according to [47]) or PancreaCult Organoid Growth Medium (OGM, Human) (Stem Cell Technologies). Organoids were propagated at 37°C under 5% (v/v) CO_2_ atmosphere and passaged for cell expansion approximately every 5–14 days depending on cell density and growth rate.

### 5.9 Cell Cytotoxicity Assay of Patient‐Derived Organoids

Prior to seeding, organoids were dissociated into single cells by dissolving the domes for 2.5 h at 37°C with 1 mg/mL Collagenase/Dispase (Roche/Merck) and afterward digested into single cells using Accutase (Sigma‐Aldrich) for 30–60 min. Cells were counted and mixed with Growth Factor Reduced (GFR) Matrigel (Corning). One μL domes containing 500 cells were dispensed per well of a white/clear bottom 384‐well plate (Brand) using a Mantis Liquid Handler (Formulatrix). Cells were cultured in 25 μL of PancreaCult OGM (Stem Cell Technologies). After 24 h, 25 μL of medium supplemented with single‐agent olaparib (0.082–50 μM), talazoparib (0.082–50 μM), PAD_ola_ or PAD_tal_ (DNA‐PKi 0.004–2.5 μM, ATRi 0.002–1.25 μM and olaparib or talazoparib (0.082–50 μM)) along with respective DMSO controls. After a 4‐day incubation, cytotoxicity was analyzed with the CytoTox‐Glo assay (Promega) according to the manufacturer's protocol. Luminescence was measured with an integration time of 1000 ms using the microplate reader Tecan Infinite M200 Pro (Tecan). Chemotherapeutics were tested at least in duplicates and values were normalized to the mean of control wells. All steps were performed by using the Mantis pipetting robot. Cell death ratio (CDR) was determined using this formula: CDR = (S1:S2) Drug/(S1:S2) Vehicle as previously described [21]. Data are represented as mean ± SD of one to three independent experiments.

### 5.10 Statistical Analysis

GraphPad Prism software was used for statistical analysis. Groups of two were analyzed with two‐tailed Student's t‐test, groups greater than two with a single variable were compared using one‐way ANOVA analysis with Tukey post hoc test, and groups greater than two with multiple variables were compared with two‐way ANOVA with Tukey post hoc test. The following values were used to denote statistical significance: *, *p* ≤ 0.05; **, *p* ≤ 0.01; ***, *p* ≤ 0.001; and ****, *p* ≤ 0.0001.

### 5.11 Study Approval

Animal procedures were approved by the Governmental Review Board of the state of Baden‐Württemberg (protocol #1369) and by the Institutional Animal Care and Use Committee of the University of California Irvine (protocol #AUP‐23‐084), respectively. Female NOD‐*scid* IL2Rgamma^null^ (NSG) mice were bred in‐house at the University of California Irvine and purchased from Jackson Laboratory. C57BL/6J mice were bred in‐house at the University of California Irvine. All mouse work aspects were carried out following strict guidelines to ensure careful, standardized, consistent, and ethical handling of mice.

## Author Contributions

A.K.B, A.K., J.G. and L.P. designed the research studies. A.K.B, M.E., C.J.H., J.L., E.R., S.C., J.G. and L.P. conducted experiments. A.K.B., C.J.H., A.K., J.G. and L.P. acquired data. C.J.H., and A.K. provided reagents and cell lines. A.K.B. and J.G. analyzed data and prepared figures. A.K.B and J.G. drafted the manuscript. C.J.H, T.S., A.K., J.G., L.P. supervised the work. A.K.B., M.E., C.J.H., A.K., J.L., E.R., S.C., T.S., A.K. J.G. and L.P. edited and revised the manuscript. All authors read and approved the final manuscript.

## Ethics Statement

The biomaterial used was provided by Ulm University Hospital following the regulations and the vote of the Ethics Committee of Ulm University (project number 72/19).

## Conflicts of Interest

T.S. reports grants and personal fees from Celgene and Sanofi, personal fees from Amgen, AstraZeneca, Bayer, the Falk Foundation, Lilly, Merck‐Serono, Merck, Pierre Fabre, Roche, Servier, and Shire, and grants from Boehringer Ingelheim outside the submitted work. A.K. reports personal fees from the Falk foundation outside the submitted work. L.P. reports travel expenses from Ipsen and AstraZeneca and declares a consulting role for AstraZeneca, Roche and Servier outside the submitted work. No disclosures were reported by the other authors.

## Supporting information

Supporting Information S1

Figure S1

Figure S2

## Data Availability

The data that support the findings of this study are available from the corresponding authors upon reasonable request.
