# Supplementary material for: Homologous Repair‐Deficient Pancreatic Cancer: Refined Targeting of DNA Damage Response is an Effective Therapeutic Strategy
Source: United European Gastroenterol J. 2025 Aug 18;13(7):1328–42. doi: 10.1002/ueg2.12773 (PMC12463693; doi:10.1002/ueg2.12773)
Supplement: Supplementary file 1 — Supporting Information S1 [file UEG2-13-1328-s001.docx]

**Supplementary Figure S1. HRD malignant cells are susceptible to DNA** **damage response interference.**

**A** and **B,** Drug viability assays of DNA-PK inhibitor (DNA-PKi CC-115) (**A**) and ATR inhibitor (ATRi VE-822) (**B**) treatment in *Kras^LSL-G12D/+^*; *Ptf1a^Cre/+^* (KC) and *Atm^fl/fl^*; *Kras^LSL-G12D/+^*; *Ptf1a^Cre/+^* (AKC) cells. **C,** Drug viability assay on KC and AKC cells treated with varying concentrations of DNA-PKi (CC-115, 0.1-2.5 µM) and ATRi (VE-822, 0.0025-1.000 µM), plus 0.005 µM or 0.025 µM olaparib or talazoparib, respectively. Solid white lines delimit the area with a cell viability below 70%. **D** and **E,** Drug viability assay of DNA-PKi (CC-115) (**D**) and ATRi (VE-822) (**E**) treatment in *Kras^LSL-G12D/+^*; *Trp53^LSL-R270H/+^*; *Pdx1*-*Cre* (KPC), *Brca1^fl/fl^*; *Kras^LSLG12D/+^*; *Trp53^LSL-R270H/+^*; *Pdx1*-*Cre* (BRCA1^KPC^), *Brca2^fl/fl^*; *Kras^LSL-G12D/+^*; *Trp53^LSL-R270H/+^*; *Pdx1-Cre* (BRCA2^KPC^), and *Palb2^fl/fl^*; *Kras^LSL-G12D/+^*; *Trp53^LSL-R270H/+^*; *Pdx1*-*Cre* (PALB2^KPC^) cells*.* **F,** Drug viability assay on KPC, BRCA1^KPC^, BRCA2^KPC^ and PALB2^KPC^ cells with varying concentrations of DNA-PKi (CC-115, 0.1-2.5 µM) and ATRi (VE-822, 0.0025-1.0000 µM), plus 0.0050 µM or 0.0025 µM olaparib or talazoparib, respectively. Solid white lines delimit the area with a cell viability below 70%.

**Supplementary Figure S2. PAD is a tolerable regimen *in vivo.***

**A** and **B,** Percentage change of NSG mouse body weight under treatment with PAD_ola_ (ATR inhibitor VE-822, 20.0 mg/kg; DNA-PK inhibitor CC-115, 2.5 mg/kg; and PARP inhibitor olaparib, 50.0 mg/kg), or PAD_tal_ (ATR inhibitor VE-822, 20.0 mg/kg; DNA-PK inhibitor CC-115, 2.5 mg/kg; and PARP inhibitor talazoparib, 0.1 mg/kg) from subcutaneous assay shown in **Figure 3A** (**A**) and from subcutaneous assay shown in **Figure 4A** (**B**). The horizontal dotted line represents 20% weight loss. **C** and **D**, Immunohistochemistry staining for H2AX p-S139 (**C**) and cleaved caspase-3 (**D**) in resected organ tissue from toxicity assay shown in **Figure 4L**. Scale bars represent 100 µm. Data are represented as mean ± SD. **E**, Quantification of H2AX p-S139-positive cells in resected intestine following treatment with PAD_ola_, and PAD_tal_, respectively. Data are represented as mean ± SD. CC3, cleaved caspase-3. One-way ANOVA with Tukey post hoc test (**E**).
