# Supplementary figures and images for: Homologous Repair‐Deficient Pancreatic Cancer: Refined Targeting of DNA Damage Response is an Effective Therapeutic Strategy
Source: United European Gastroenterol J. 2025 Aug 18;13(7):1328–42. doi: 10.1002/ueg2.12773 (PMC12463693; doi:10.1002/ueg2.12773)

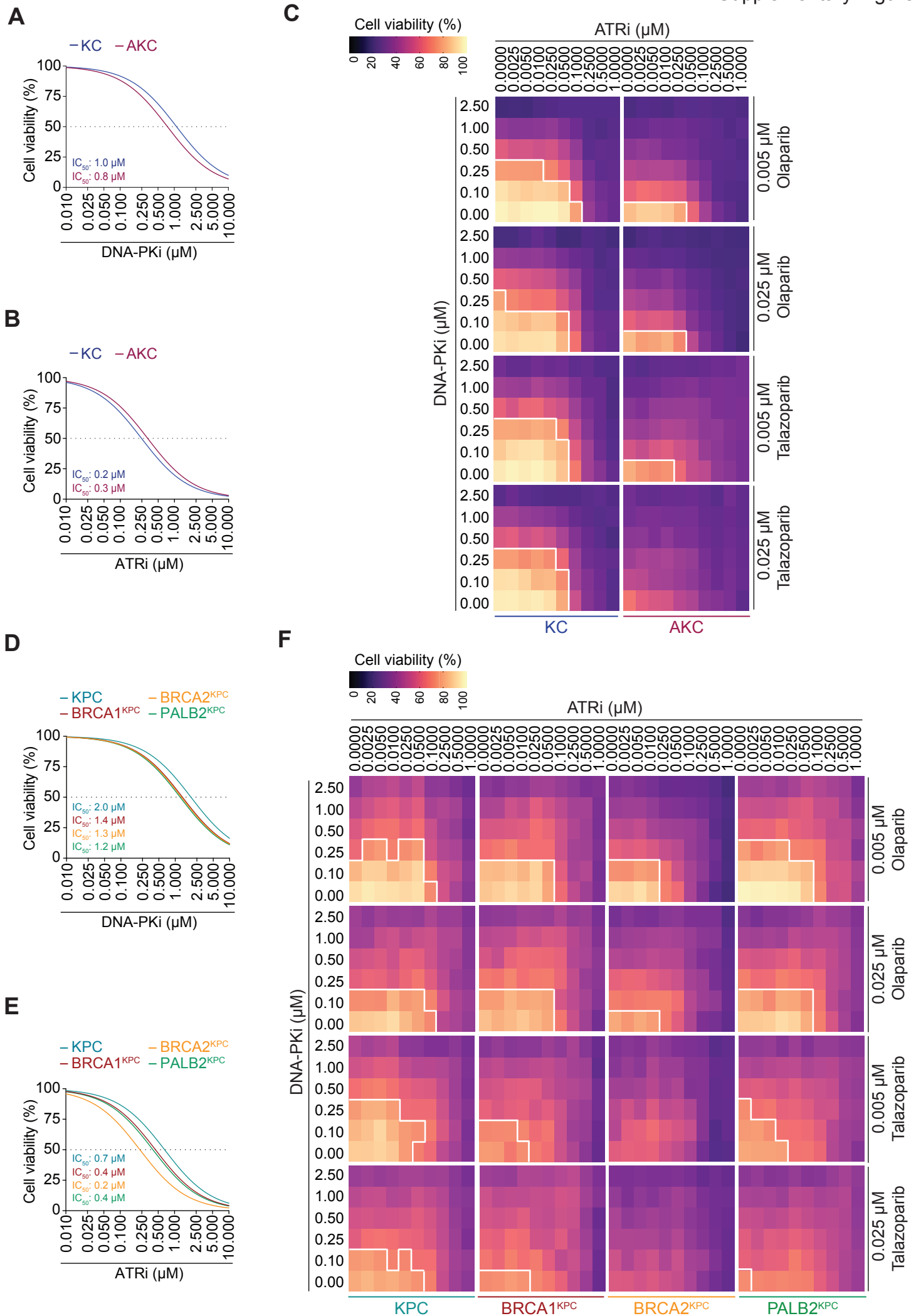

Supplement: Supplementary file 2 — Figure S1 [file UEG2-13-1328-s003.pdf]

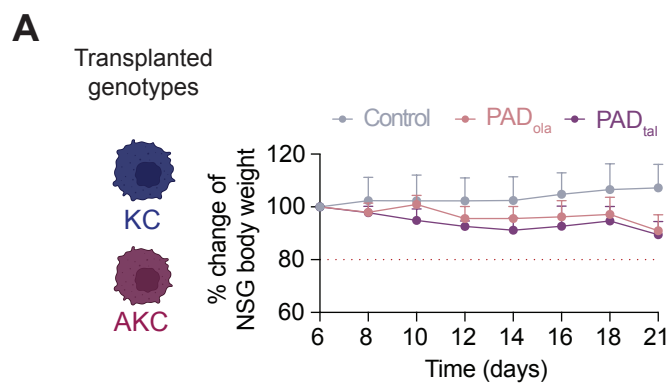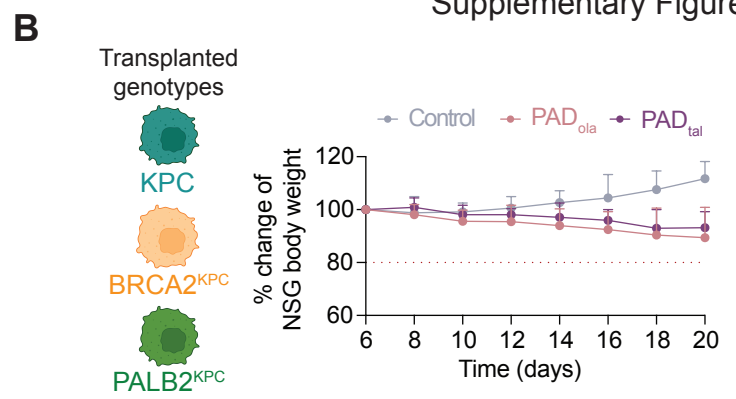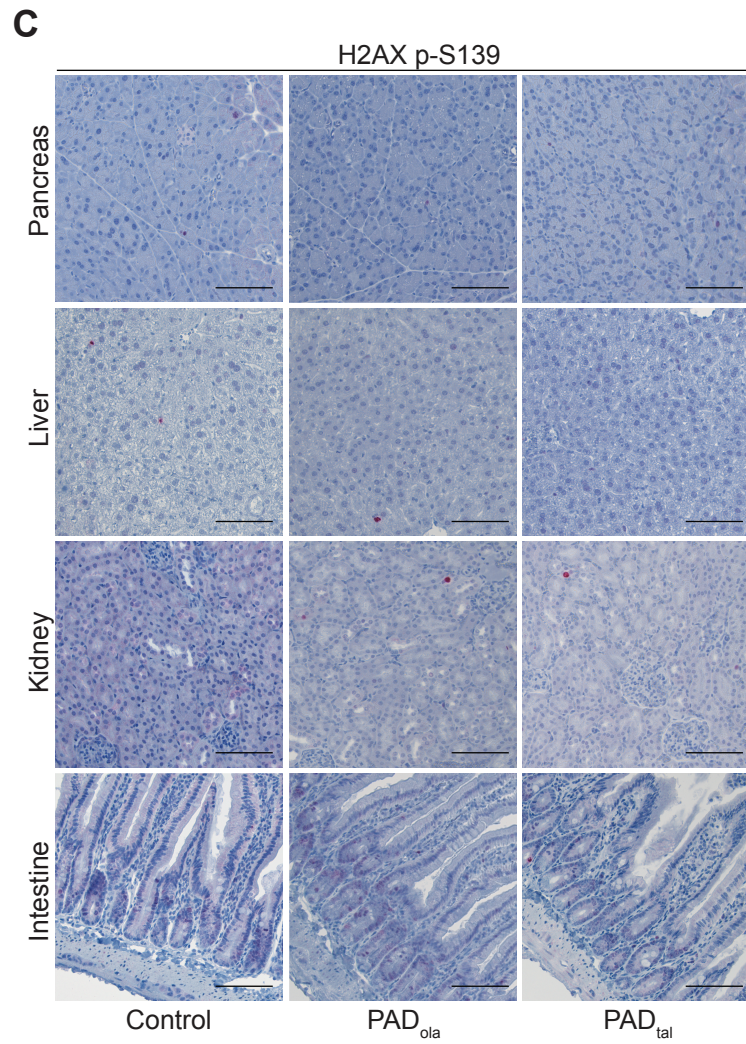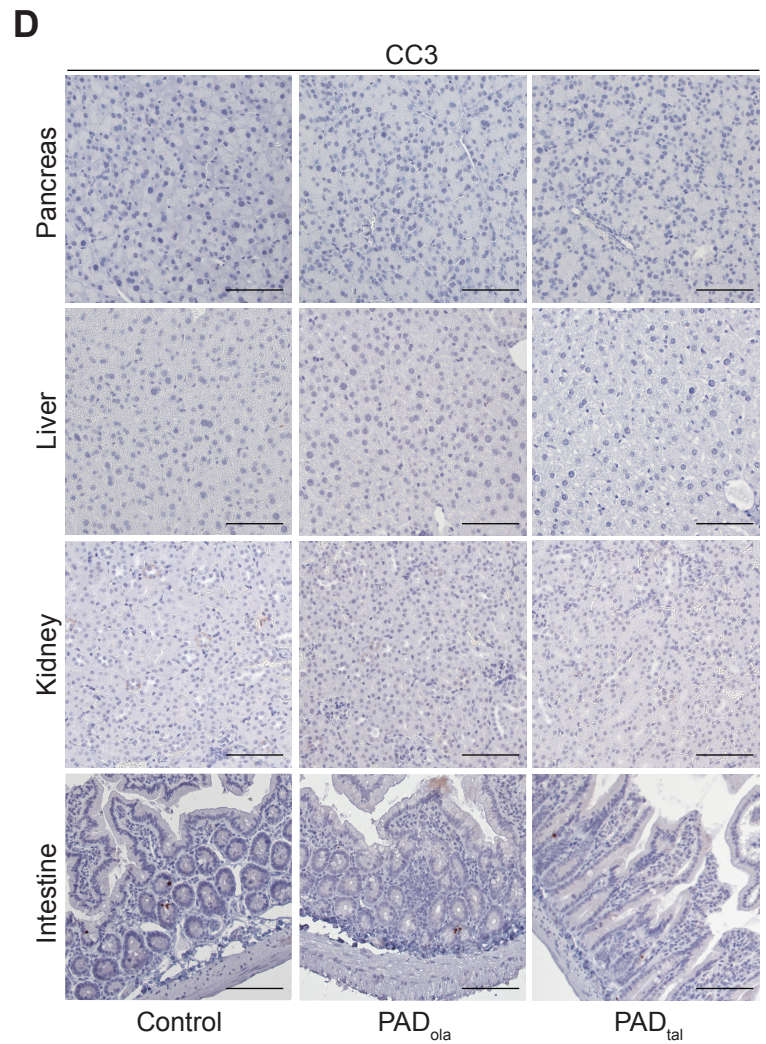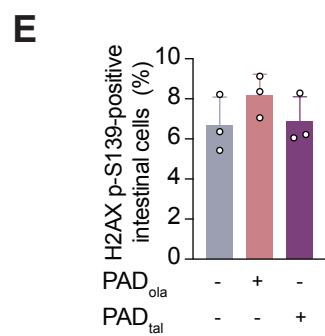

Supplement: Supplementary file 3 — Figure S2 [file UEG2-13-1328-s002.pdf]
